# Fly ash boosted electrocatalytic properties of PEDOT:PSS counter electrodes for the triiodide reduction in dye-sensitized solar cells

**DOI:** 10.1038/s41598-023-33020-6

**Published:** 2023-04-12

**Authors:** Nattakan Kanjana, Wasan Maiaugree, Paveena Laokul, Inthira Chaiya, Thodsaphon Lunnoo, Poramed Wongjom, Yingyot Infahsaeng, Bunjong Thongdang, Vittaya Amornkitbamrung

**Affiliations:** 1 Thammasat University Research Unit in Energy Innovations and Modern Physics (EIMP), Thammasat University, Pathum Thani, 12120 Thailand; 2 Division of Physics, Faculty of Science and Technology, Thammasat University, Pathum Thani, 12120 Thailand; 3 Department of Physics, Faculty of Science, Mahasarakham University, Kantarawichai, Mahasarakham 44150 Thailand; 4 Department of Mathematics, Faculty of Science, Mahasarakham University, Kantarawichai, Mahasarakham 44150 Thailand; 5 Thammasat University Research Unit in Quantum Technology, Thammasat University, Pathum Thani, 12120 Thailand; 6 Electricity Generating Authority of Thailand, Nonthaburi, 11130 Thailand; 7 Thailand Center of Excellence in Physics (ThEP center), Ministry of Higher Education, Science, Research and Innovation, Bangkok, 10400 Thailand

**Keywords:** Energy science and technology, Materials science, Materials for energy and catalysis, Physics

## Abstract

Fly ash solid waste from a power plant was applied in a solar cell application for the first time. A doctor blade was used to coat FTO-glass with a composite film of mixed fly ash and PEDOT:PSS (FP). XRD, FTIR, SEM, EDX, and BET analyses were used to elucidate the crystal structure, morphology, and functional groups of fly ash in the current research. A significantly high efficiency solar cell was fabricated utilizing fly ash. CV, Tafel, and EIS analyses indicated a decrease in charge transfer resistance and an increased catalytic activity in the counter electrodes. The performance of DSSCs made from FP counter electrodes varied depending on the percentage of fly ash particles present. Fly ash mixed with PEDOT:PSS in a concentration ratio of 2:5 g/mL showed a high efficiency of 4.23%, which is comparable to Pt DSSC's (4.84%). Moreover, FP-2:5 presented a more highly efficient electrode than counter electrodes made from PEDOT:PSS mixed with MoO (3.08%) and CoO (3.65%). This suitability of this low-cost CE material for use in DSSCs has been established.

## Introduction

Dye-sensitized solar cells (DSSCs), an alternative form of planner silicon solar cells, have been extensively explored due to their low cost, ease of fabrication, simplicity and design versatility. In DSSCs, the working electrode (WE) is typically formed from porous titanium dioxide nanoparticles, with N719 dye as the sensitizer, an iodide (${\text{I}}^{ - }$)/triiodide (${\text{I}}_{3}^{ - }$) redox electrolyte, and platinum (Pt) as the counter electrode (CE)^1–4^.

Currently, the goal of developing DSSCs is to achieve high efficiency and to reduce production costs, which are new challenges for researchers. Carbon black^5^, multi-walled carbon nanotubes^6^, conductive polymers^7^, active carbon^8^, and metal oxide materials^9,10^ as well as sulfide materials^11^ have been used to replace expensive catalytic Pt because of their high electrical conductivity, chemical resistance, electrocatalytic properties and low cost. Our research focuses on the development of novel Pt-free CE materials that are both low-cost and environmentally friendly, as well as consideration of waste recycling by including solid waste from power plants. In Lampang Province of Northern Thailand, the Mae Moh power plant uses great quantities of coal as a source of energy. This results in large amounts of solid waste in the form of fly ash, bottom ash and gypsum. However, bottom ash and gypsum quantities are normally much lower than that of fly ash^12^. Developing removal methods or effective exploitation of fly ash has been a challenge of researchers. At present, fly ash is primarily used as a raw material for cement, concrete production and soil augmentation due to its excellent adhesion, corrosion resistance and compressive strength^13,14^. Alternatively, there is no research that has applied fly ash from a power plant for use in solar cells. This may be due to its poor conductivity. However, its high electrolyte corrosion resistance and ease in forming bonds makes it an attractive material. Moreover, it has been reported that using fly ash as a composite material can enhance stability and electrocatalytic properties. For instance, according to Thirumalai et al.^15^ fly ash loaded zinc oxide exhibits higher electrocatalytic activity and electrochemical stability than bare ZnO catalyst due to its increased effective surface area. Additionally, fly ash and TiO_2_ nanocomposites (FA-TiO_2_) developed by Altalhi et al.^16^ demonstrated superior electrochemical catalytic performance for the hydrogen evolution reaction (HER) in an alkaline solution. As a result, the FA-TiO_2_ catalyst as prepared has more active catalytic sites that can produce H_2_ and cathodic activation. Therefore, the higher electrochemical and stability properties of fly ash-based catalysts are critical in their DSSC applications. One approach to improve the properties of fly ash is to composite it with conductive polymers^17^. Many previous studies reported on the use of conductive polymers for CEs in DSSCs such as poly(3,4-ethylenedioxythiophene) (PEDOT)^18^, polypyrrole (PPy)^19^, poly(3,4-ethylenedioxythiophene):polystyrene sulfonate (PEDOT:PSS)^20^, polyvinylidene fluoride (PVDF)^21^ or poly(vinyl pyrrolidone)/polyaniline (PVP/PANI)^22^. These polymers present good counter electrode properties for DSSCs. To improve the electrocatalytic activity of CEs, nanoparticles and microparticles of metal oxides or non -metal oxides were added to the conductive polymer films in other early research studies^23,24^. Maiaugree et al.^25^ developed a counter electrode consisting of TiO_2_ mixed with PEDOT:PSS in 2012. It was applied using a doctor blade, yielding a high efficiency of 8.49%, comparable to a Pt CE (7.50%), as TiO_2_ nanoparticles can increase polymer surface for enhancing film redox reactions. A CE with a high efficiency of 6.50% (Pt CE was 6.48%) was created by Xu et al.^26^ using a TiO_2_/SnO_2_ a nanoporous composite film combined with PEDOT:PSS. Owing to its more active sites for ${\text{I}}_{3}^{ - }$ reduction, the obtained TiO_2_/SnO_2_/PEDOT:PSS composite film exhibits better catalytic activity for triiodide reduction than the pristine PEDOT:PSS film. This leads to the material's fill factor and efficiency of the cells being significantly increased. Porous rGO/ZnSe/CoSe_2_ combined with a PEDOT:PSS counter electrode created by Tapa et al.^27^ had a high solar cell efficiency of 8.60% and outperformed platinized CEs (7.14%) in terms of catalytic activity for the ${\text{I}}^{ - }   /{\text{I}}_{3}^{ - }$ redox reaction. Ahmed et al.^28^ created a MoS_2_/NC composite with PEDOT:PSS. The PCE of the device with a MoS_2_/NC-PEDOT:PSS composite CE with larger surface area was 7.67% higher, resulting in higher electrocatalytic performance than the bare PEDOT:PSS CEs (4.11%). Recently, Our research employed agricultural residue activated carbon combined with PEDOT:PSS with a DSSC efficiency of 5.85%, higher electrocatalytic activities for ${\text{I}}_{3}^{ - }$ reduction, conductivity and higher efficiencies than Pt CEs (5.43%)^29^. Thus, mixing fly ash into PEDOT:PSS may increase the number of active sites, catalytic properties and efficiency of DSSCs.

Normally, many agricultural residues were promoted as recycling materials for counter electrodes in DSSCs. However, the sources of these materials are confined only to biomass or carbon-based materials. So, this work will present a new waste material from non-biomass or noncarbon based materials that are the residues of the combustion of coal for counter electrodes. This is because biomass materials must be surface treated and carbonized prior to use, which adds to the process and cost, as well as the complexity of CE fabrication. Based on these constraints, the researchers chose non-biomass materials for their studies in order to reduce the number of steps and complexity of producing CEs, while also developing a method to use this material.

In this paper, we propose a novel counter electrode for DSSCs made of fly ash mixed with PEDOT:PSS (FP) in a composite film. Fly ash serves as a material for increasing the number of active sites, while PEDOT:PSS acts as a catalyst and binder in the FP film. Additionally, a quick, low-cost, single-step fabrication procedure is provided. According to these findings, the performance of DSSCs made from FP films varied depending on the percentage of incorporated fly ash particles. Fly ash mixed with PEDOT:PSS in a concentration ratio of 2:5 g/mL yielded a high efficiency (4.23%). This efficiency approaches that of Pt DSSC's (4.84%). This work presents a highly efficient electrode fabricated from fly ash incorporation that has enhanced electrocatalytic activity and solar cell performance comparable to that of electrodes made using MoO and CoO.

## Experimental

### Preparation of fly ash counter electrodes

**Figure 1:**
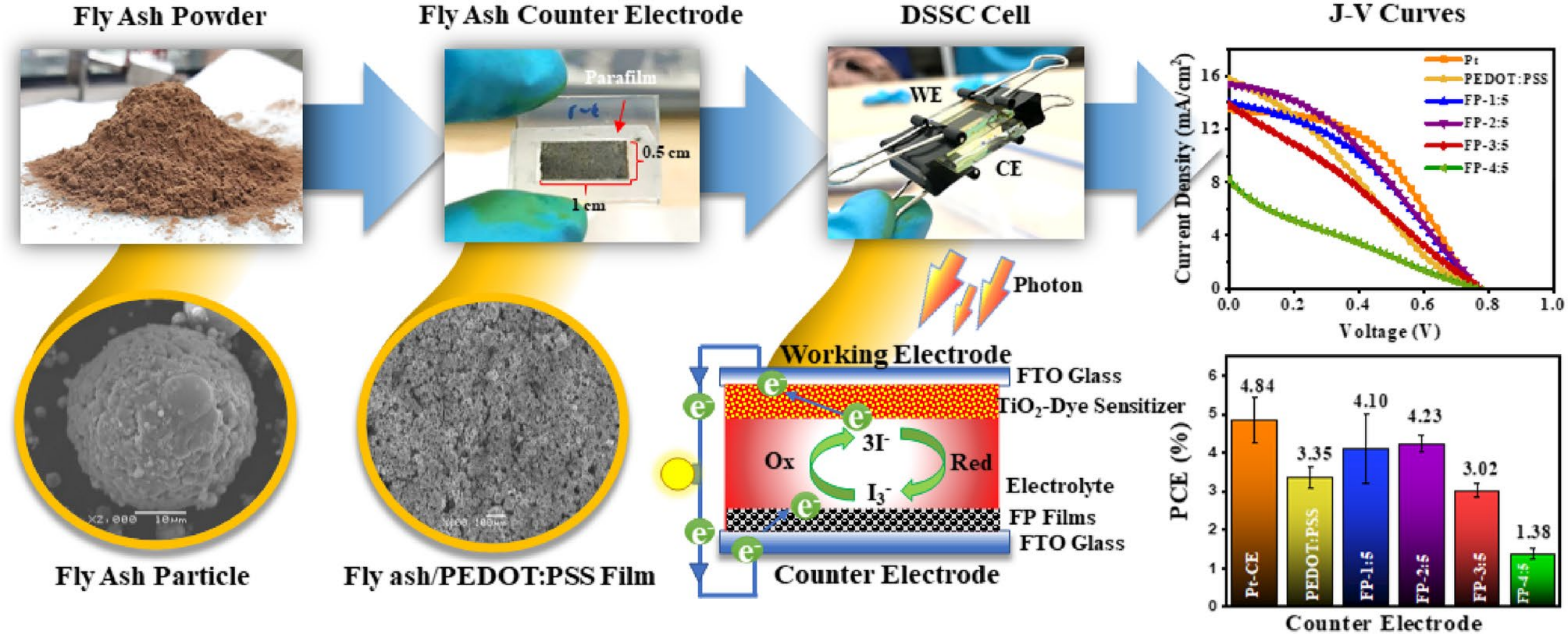
Schematic of the fly ash counter electrode and DSSC J-V curves.

The Mae Moh Power Plant in Lampang Province of Northern Thailand was the source of the fly ash powder used in the current study. It was heated at 80 °C for 24 h. PEDOT:PSS (Sigma Aldrich) was dissolved in distilled water in a mass/volume ratio of 1 to 1. Fly ash was added and mixed in various ratios of fly ash to PEDOT:PSS of 1:5, 2:5, 3:5, and 4:5 g/mL. These samples are referred to as FP-1:5, FP-2:5, FP-3:5 and FP-4:5, respectively. The FP mixtures were stirred for 15 min. Fluorine-doped tin oxide glass (FTO, 15/sq, Solaronix) was thoroughly cleaned with soapy water, followed by 30 min of ultrasonication with deionized water and ethanol. After drying, FP films were prepared and applied to FTO glass over a 0.5 cm^2^ area masked with tape using a doctor blade (Fig. 1). Then, the obtained FP films were heated at 80 °C for 6 h. Using Pt as a CE standard, Pt electrode was prepared by mixing 3 mM tetraammineplatinum(II) chloride hydrate (98%, Sigma Aldrich) and 0.2 g ethyl cellulose (Sigma Aldrich) in isopropyl alcohol (Sigma Aldrich) and spin-coated at 500 rpm for 30 s and 1500 rpm for 30 s followed by drying at 80 °C onto FTO glass for three cycles before sintering at 500 °C for 1 h under an air atmosphere.

### Fabrication of DSSCs

The working electrodes were made by screen-printing TiO_2_ PST-18NR paste (Solaronix) onto an FTO substrate with a film thickness of 16 μm with no blocking layer and scattering layer. After annealing at 500 °C for 60 min under an air atmosphere, the TiO_2_ photoanodes were immersed in N719 dye at a concentration of 0.5 mM for 24 h. Previous work^4^ was used to guide the manufacture of the N719 dye solution (Solaronix S.A.) and the ${\text{I}}^{ - }   /{\text{I}}_{3}^{ - }$ electrolyte used in this study. N719 dye was prepared using 0.5 mM ruthenizer 535-bisTBA (solaronix) and 50 ml tert-Butanol (> 99.70%, Sigma Aldrich) in 50 ml acetonitrile (99.8%, LiChrosolv). An ${\text{I}}^{ - }   /{\text{I}}_{3}^{ - }$ electrolyte was prepared using 0.1 M lithium iodide anhydrous (99.99%, Alfa Aesar), 0.05 M iodine (> 99.8%, Riedel–de Haen), 0.6 M 1-propyl-3-methylimidazolium iodide (> 98%, Sigma-Aldrich), 2.5 mM lithium carbonate (99.99%, Sigma-Aldrich) and 0.5 M tert-butylpyridine (96%, Sigma-Aldrich Corp.) in acetonitrile.

A semi-closed approach was used to assemble the WE and CE with a parafilm as a separator and a drop of ${\text{I}}^{ - }   /{\text{I}}_{3}^{ - }$ electrolyte for the DSSC asymmetrical cell assembly (Fig. 1). In the case of the CE:CE symmetric cells, two FA CEs with an active area of 0.5 cm^2^ were assembled using a parafilm separator and then filled with the same ${\text{I}}^{ - }   /{\text{I}}_{3}^{ - }$ electrolyte to perform EIS and Tafel tests on the CE catalysts.

### Characterizations

The crystal structure and functional groups of fly ash were examined using XRD patterns (Bruker D8 Advance X-ray Diffractometer (Cu-Kα source)), and Fourier transform infrared spectroscopy (FT-IR, Bruker UV-1900). The optical absorption spectrum of fly ash, PEDOT:PSS and fly ash mixed PEDOT:PSS suspensions were examined using fluorescence and absorbance spectrometer (Duetta, Horiba) over the entire wavelength range from 250 to 1100 nm, in which samples were prepared by dispersing the sample powder in an ethanol solution. To characterize morphology, scanning electron microscopy (SEM, LEO-1450VP, UK) was applied while energy dispersive X-ray spectroscopy (EDX, Hitachi TM4000Plus) was used to determine the elemental composition of the fly ash. The specific surface area and pore volume of fly ash were calculated using the Brunauer–Emmett–Teller (BET) and Barrett–Joyner–Halenda (BJH) methods, measuring the nitrogen adsorption–desorption isotherm. The porosity, total pore area and average pore diameter of fly ash were examined using a Mercury Intrusion Porosimeter (Micromeritics AutoPore V 9600).

To determine the electrochemical activity of the FA CEs, the catalytic activity of the counter electrode was measured in a three-compartment cell using cyclic voltammetry (CV, Gamry REF 3000, U.S.A) at a scan rate of 20 mV/s. A Pt plate was used as a counter electrode, and Ag/AgCl was employed as a reference electrode. The CV electrolyte in the three-electrode system was prepared using 10 mM lithium iodide anhydrous, 1 mM iodine and 0.1 M lithium perchlorate anhydrous (99%, Alfa Aesar) in acetonitrile.

A solar simulator (Peccell, PE-L111, Japan) system with a light intensity of 100 mW/cm^2^ was employed to study the performance of the solar cells. DSSC asymmetrical cell and CE:CE symmetrical cell impedance was measured using electrochemical impedance spectroscopy (EIS, Gamry REF 3000, USA) with a light intensity of 100 mW/cm^2^ and under a dark condition, respectively, using an AC amplitude of 10 mV over a frequency range of 0.2 Hz to 100,000 Hz.

Tafel polarization was also measured in CE:CE asymmetrical cells under a dark condition and using the same ${\text{I}}^{ - }   /{\text{I}}_{3}^{ - }$ electrolyte and measurement conditions as the symmetric CE:CE symmetrical cell impedance measurement. The measured impedance spectra were matched with Gamry Echem Analyst software using equivalent circuit model.

## Results and discussion

**Figure 2:**
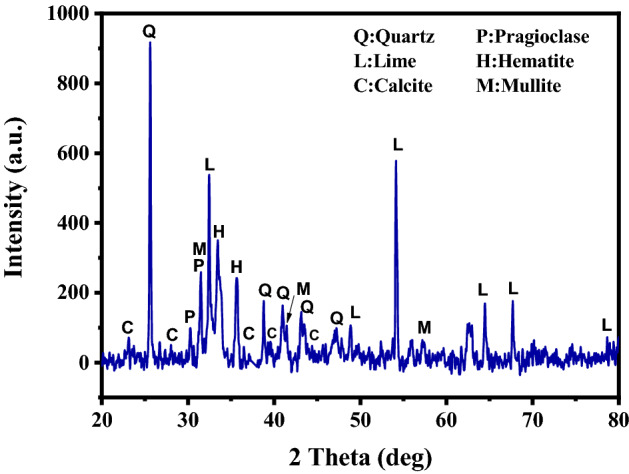
XRD pattern of fly ash annealed at 80 °C.

**Figure 3:**
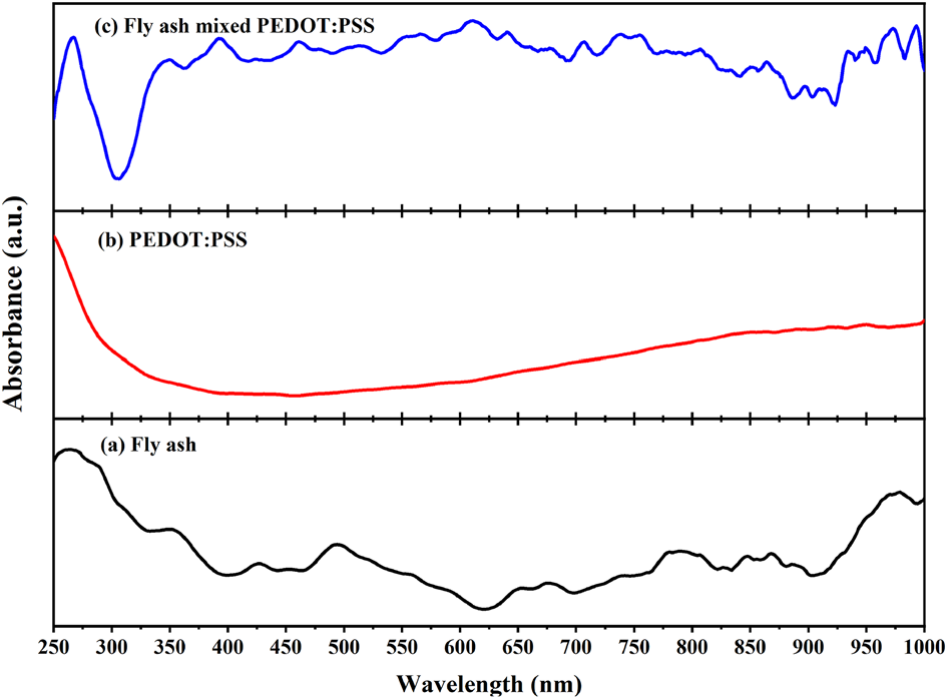
The UV–vis spectrum of (**a**) fly ash, (**b**) PEDOT:PSS, and (**c**) fly ash mixed with PEDOT:PSS.

Figure 2 presents XRD pattern of fly ash. The material was found to contain crystalline phases such as those of quartz (SiO_2_), lime (CaO), calcite (CaCO_3_), pragioclase (NaAlSi_3_O_8_–CaAl_2_Si_2_O_8_), hematite (Fe_2_O_3_), and mullite (Al_6_Si_2_O_13_)^30–33^. However, other heavy metals were not observed presumably due to their lower quantities in fly ash, which were below the XRD detection limit. The UV–vis spectrum of fly ash is depicted in Fig. 3a. It was also discovered that fly ash has a wide light absorption range from ultraviolet (UV) to near-infrared (NIR) light. The absorption of UV light in the wavelength range from 250 to 400 nm is the highest compared to that in other regions. This may be a result of the major constituents present in the fly ash, mostly SiO_2_ based on XRD result, whose energy bandgap corresponds to absorbance in the UV range^34^. While the absorption spectrum of PEDOT:PSS (Fig. 3b) exhibits wide absorption characteristics in the UV and NIR region, the literature shows that PEDOT:PSS has a main absorption peak at wavelength about 224 and 254 nm. The two absorption bands corresponding to the aromatic ring of a –PSS group^35,36^, but the main peaks at these positions are not shown as a result of the limitation of the instrument with an initial wavelength limit of 250 nm. However, when considering the absorbance in the infrared region from 800 to 1200 nm of PEDOT:PSS, it was found that the absorption behavior in this region was consistent with Kim J. et al.'s research^37^. Compared with PEDOT:PSS, fly ash mixed PEDOT:PSS shows an increased absorbance in the wavelength range from 300 to 1000 nm, as shown in Fig. 3c. This can confirm the possibility of intermolecular interactions between fly ash and PEDOT:PSS.

**Figure 4:**
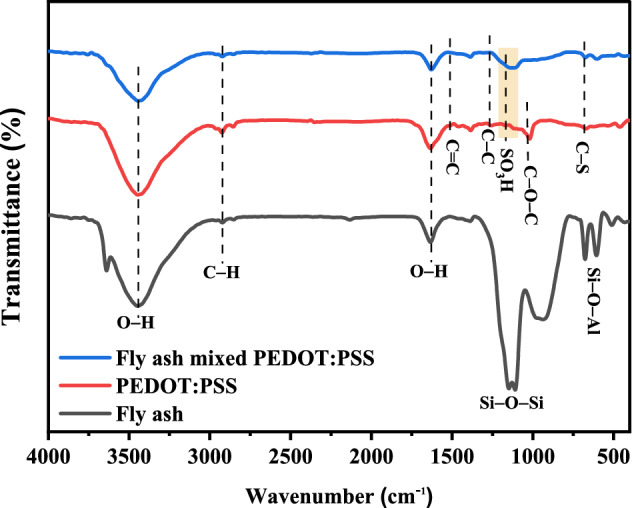
FTIR spectra of fly ash annealed at 80 °C, PEDOT:PSS, and fly ash mixed with PEDOT:PSS powder.

The FTIR spectra of fly ash, PEDOT:PSS, and fly ash mixed PEDOT:PSS powders are shown in Fig. 4. The signals originating at 3442 cm^−1^ and 2923 cm^−1^ correspond to –OH groups and C–H stretching. The presence of –Si–OH groups and water absorbed in the fly ash are related to the bands at 1634 cm^−1^^38,39^. The Si–O–Si antisymmetric stretching mode of silica is related to the band at 1100 cm^−1^, while the Si–O–Al stretching of fly ash was observed at 605 cm^−1^^38^, respectively. PEDOT:PSS and fly ash mixed PEDOT:PSS exhibit similar FTIR bands. The C–C and C=C stretching modes of the quinoid structure in thiophene rings are represented by the spikes at 1290 and 1518 cm^−1^, respectively, whereas the peaks at 1135 cm^−1^ reveal C–O–C stretching^36,40,41^. Furthermore, the spikes at 1201 and 824 cm^−1^ correlate to the stretching vibrations of the SO_3_H group of PSS and C–S bonds in the thiophene rings of PEDOT^41,42^. When compared to PEDOT:PSS, the intensity of the fly ash mixed PEDOT:PSS sample is less and the absorption peaks are slightly shifted. These findings suggest the possibility of intermolecular interactions between fly ash and PEDOT:PSS. The results are consistent with Xu et al.^41^ who prepared a nanocomposite film based on PEDOT:PSS modified with dual additives of carbon black (CB) and dimethyl sulfoxide (DMSO). It was found that in addition to the peak intensity weakening, several absorption peaks also slightly shift. These findings imply that the CB and PEDOT:PSS interact with one another intramolecularly ($\pi$-$\pi$ interactions).

**Figure 5:**
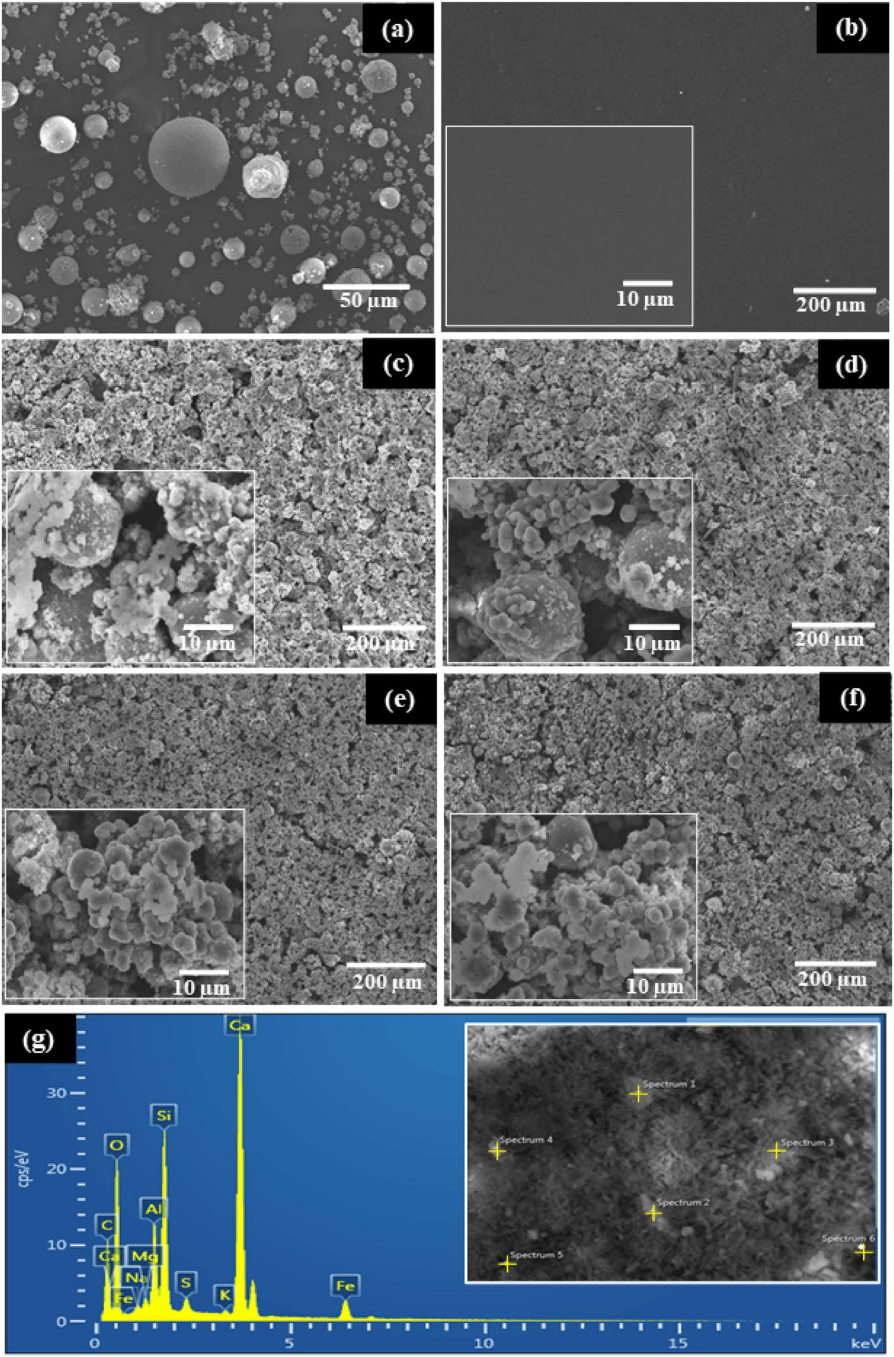
SEM images of fly ash (**a**), PEDOT:PSS film (**b**), and fly ash mixed PEDOT:PSS films (FP) with various concentration ratio of 1:5 (**c**), 2:5 (**d**), 3:5 (**e**), 4:5 g/mL (**f**) and (**g**) an EDX spectrum of fly ash.

The morphology of a fly ash particles is shown in Fig. 5a. The particle sizes ranged from 5 to 40 µm (Fig. S1). Smooth surfaces after dispersing PEDOT:PSS over the FTO substrate are shown in Fig. 5b. Furthermore, the particle morphologies include solid spheres and irregularly shaped particles. The surfaces of fly ash mixed PEDOT:PSS films with varying fly ash contents are shown in Fig. 5c–f. Formation of nanoporous and macroporous structured deposits on the fly ash surface coating is also apparent at lower magnification. At higher magnification, the texture of the film surfaces seems to be the size of fly ash particles with PEDOT:PSS deposition onto the fly ash particle surfaces. The size of the fly ash particles ranges from a few microns to tens of microns. The electrode's porous surfaces result in a large surface area and good catalytic activity.

Additionally, the morphology of microparticles and microflakes can be found in CoO and MoO, respectively, as shown in Fig. S2. CoO and MoO mixed PEDOT:PSS composite film counter electrodes are shown in Fig. S3. It can be seen that the CoO CE has a clustered dispersion of microparticles. This could be due to particle agglomeration after mixing PEDOT:PSS (Fig. S3a,a1). Concurrently, MoO microflakes have a densely packed morphology with aggregations of nanoflakes. This may result in lower porosity than in other counter electrodes (Fig. S3b,b1)).

The elemental composition of the fly ash sample is shown in Fig. 5g. The identified elements were O (34.80 ± 5.15 wt%), C (25.10 ± 4.57 wt%), Ca (16.57 ± 3.10 wt%), Fe (8.42 ± 4.84 wt%), Si (7.50 ± 2.21 wt%), Al (4.25 ± 1.63 wt%), S (1.60 ± 1.70 wt%), Mg (0.72 ± 0.40 wt%), K (0.67 ± 0.40 wt%), and Na (0.42 ± 0.09 wt%). The results of these tests are consistent with XRD analysis that showed the presence of the crystal structures of CaO, Fe_2_O_3_, SiO_2_, Al_2_O_3_, and SO_3_, as well as MgO and other substances^12,31^. However, the high carbon level may have been caused by the carbon tape used in the test.

**Figure 6:**
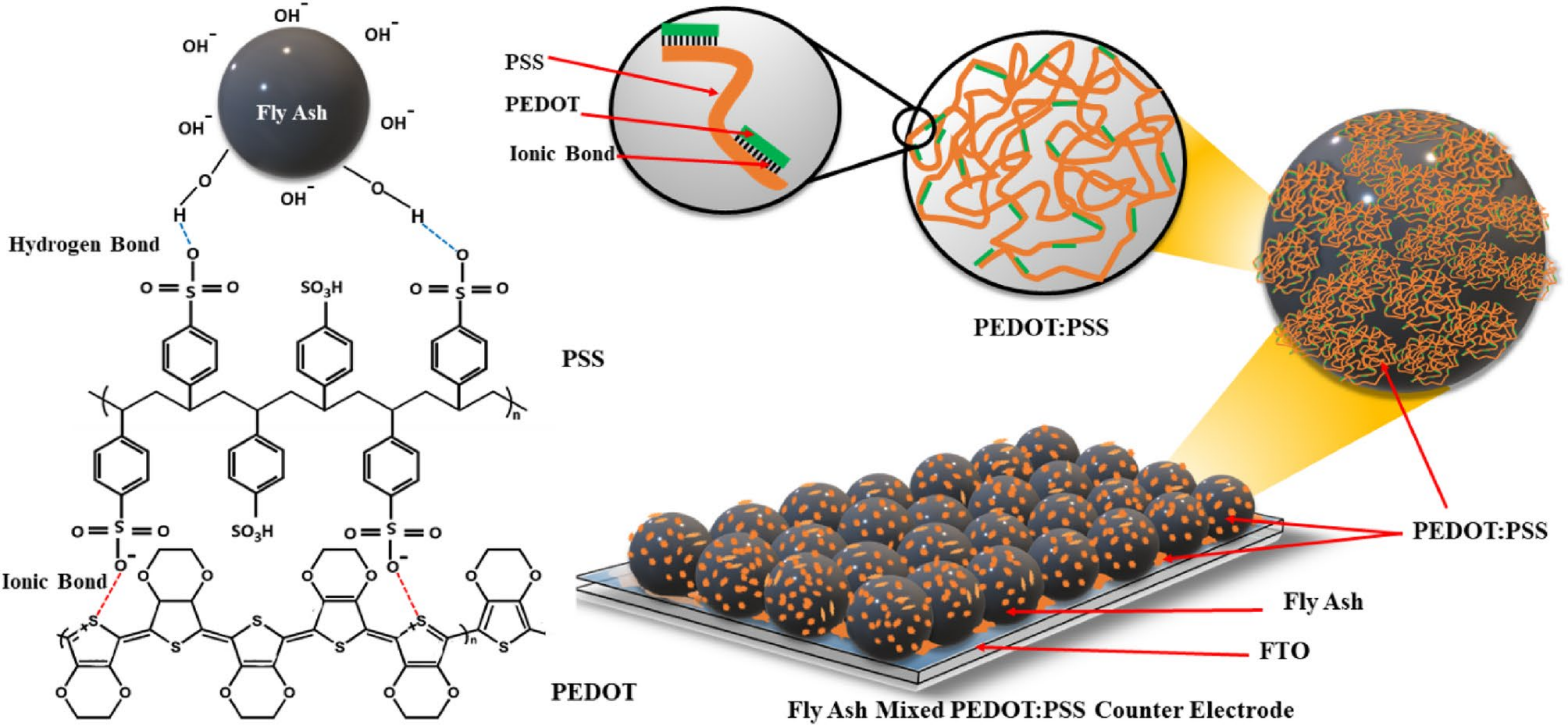
Schematic of the proposed fly ash surfaces coated with PEDOT: PSS.

The chemical bonds between PEDOT:PSS and fly ash are depicted in Fig. 6. PEDOT:PSS consists of two ionically bound polymers: PSS benzene rings containing both neutral SO_3_H and anionic SO_3_^−^, and every three or four thiophene units on PEDOT chains carries a positive charge^41–43^. Thus, a complex structure of PEDOT with PSS was created by ionic bonding between SO_3_^-^ and thiophene rings^44–46^. Moreover, a hydrogen bond was formed between the OH groups of the PSS structure and the O of the metal oxide in fly ash^47^. Additionally, PEDOT:PSS adheres to the surface of fly ash particles and can be used as a binder to join the particles to the FTO substrate and to each other, as shown in Fig. 6.

**Figure 7:**
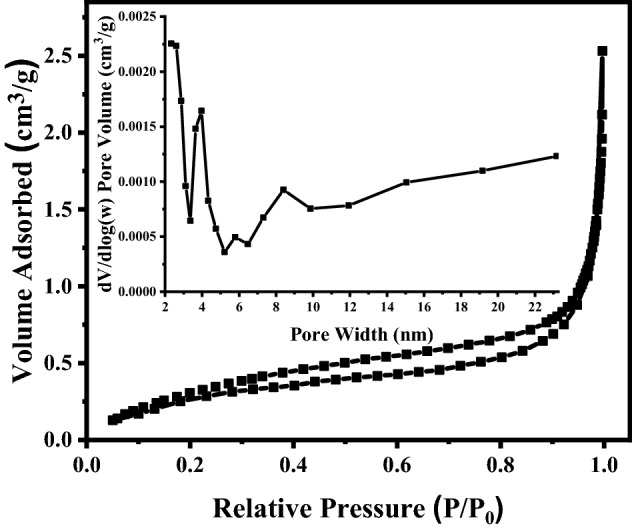
BET curves and pore size distribution (figure inset) of fly ash.

Nitrogen adsorption–desorption isotherms of fly ash are shown in Fig. 7. They exhibit a Type IV isotherm indicating a blend of microporous and mesoporous materials^48^. The surface area of fly ash was 1.439 m^2^/g^1^ according to BET analysis, while pore volume diameter was 0.0029 cm^3^/g^1^, according to BJH pore size analyses. Moreover, the cumulative intrusion curves and pore size distribution of fly ash measured from the mercury intrusion porosimetry (MIP) technique are shown in Fig. S4. The MIP results for fly ash present the porosity, total pore area and average pore diameter values of 61.07%, 3.17 m^2^/g^1^ and 0.87 µm respectively.

**Figure 8:**
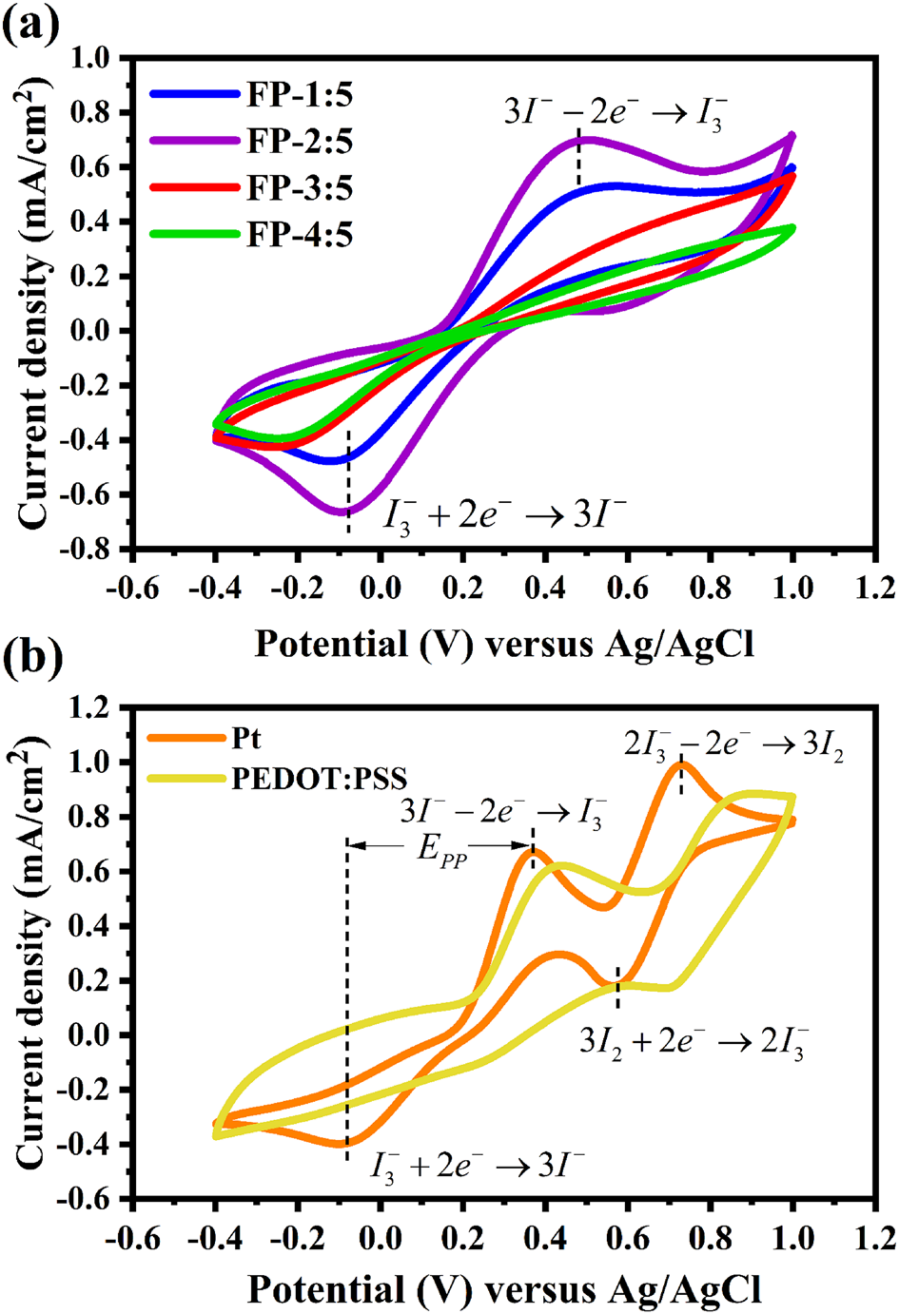
CV curves of (**a**) FP, (**b**) Pt and PEDOT:PSS counter electrodes.

Cyclic voltammetry analysis was used to study the electrochemical properties of counter electrodes using a three-electrode system consisting of FP, PEDOT:PSS and Pt. Figure 8a presents the CV curves of the FP counter electrodes. The oxidation ($3{\text{I}}^{ - }  - 2e^{ - }  \to {\text{I}}_{3}^{ - }$) and reduction (${\text{I}}_{3}^{ - }  + 2e^{ - }  \to 3{\text{I}}^{ - }$) peaks were found to have similar features. The CV curves of the PEDOT:PSS and Pt counter electrodes exhibited two pairs of redox peaks during oxidation (Ox1: $3 {\text{I}}^{ - }  - 2e^{ - }  \to {\text{I}}_{3}^{ - }$ and Ox2: $2{\text{I}}_{3}^{ - }  - 2e^{ - }  \to 3{\text{I}}_{2}$) and reduction (Red1:${\text{I}}_{3}^{ - }  + 2e^{ - }  \to 3{\text{I}}^{ - }$ and Red2: $3{\text{I}}_{2}  + 2e^{ - }  \to 2{\text{I}}_{3}^{ - }$) peaks (Fig. 8b). In a DSSC, the redox peak of the Ox1 and Red1 are focused. The redox reaction of ${\text{I}}^{ - } /{\text{I}}_{3}^{ - }$ can be investigated from the cathodic peak current ($J_{pc}$) and peak to peak voltage separation ($E_{pp}$), as two essential factors for assessing the catalytic activity of a CE. Higher $J_{pc}$ and lower $E_{pp}$ values indicates that the catalyst has superior electrochemical catalytic activity and the high oxidation–reduction reaction between ${\text{I}}^{ - }$ and ${\text{I}}_{3}^{ - }$ can occur smoothly on the catalysts' surfaces, respectively^49–52^. The $E_{pp}$ value is calculated from Eq. (1).

1 $$  E_{{pp}}  = \left| {{\text{Ep(anodic)}} - \left. {{\text{Ep(cathodic)}}} \right|} \right.  $$

The $E_{pp}$ and $J_{pc}$ values are summarized in Table 1. When comparing the FP-1:5 (0.66 V and − 0.55 mA/cm^2^), FP-3:5 (0.81 V and − 0.21 mA/cm^2^), FP-4:5 (0.88 V and − 0.19 mA/cm^2^), and PEDOT:PSS (0.62 V and − 0.31 mA/cm^2^) electrodes with the FP-2:5 electrode, the latter has a lower $E_{pp}$ and higher $J_{pc}$ (0.59 V and − 0.76 mA/cm^2^), demonstrating a superior electrochemical catalytic activity of the FP-2:5 CE. This result confirms that the introduction fly ash into PEDOT:PSS can accelerate the catalytic process of ${\text{I}}_{3}^{ - }$ reduction. Pt electrodes have an $E_{pp}$ value of 0.46 V and a $J_{pc}$ value of − 0.73 mA/cm^2^. However, the catalytic process decreased when the concentration ratio of fly ash to PEDOT:PSS was increased to more than 2:5 g/mL. This is in strong agreement with the Tafel and EIS results, as evidenced by the exchange current density ($J_{0}$) value and charge transfer resistance. These behaviors led to an increased power conversion efficiency.

**Table 1:** Summary of peak to peak voltage separation ($E_{pp}$), cathodic peak current ($J_{pc}$), diffusion-limited current ($J_{\lim }$), exchange current density ($J_{0}$) and slope of the oxidation of Ip–υ^1/2^ curves.

Counter electrode	$E_{pp}$ (V)	$J_{PC}$ (mA/cm^2^)	$J_{\lim }$(mA/cm^2^)	$J_{0}$(mA/cm^2^)	CV slope (μA/cm^2^•s^1/2^ V^1/2^)
Pt	0.46	−0.73	20.74	1.60	N/A
PEDOT:PSS	0.62	−0.31	6.32	0.11	21.10
FP-1:5	0.66	−0.55	8.94	0.30	24.61
FP-2:5	0.59	−0.76	12.17	0.38	27.75
FP-3:5	0.81	−0.21	2.56	0.05	28.96
FP-4:5	0.88	−0.19	2.26	0.04	30.48

**Figure 9:**
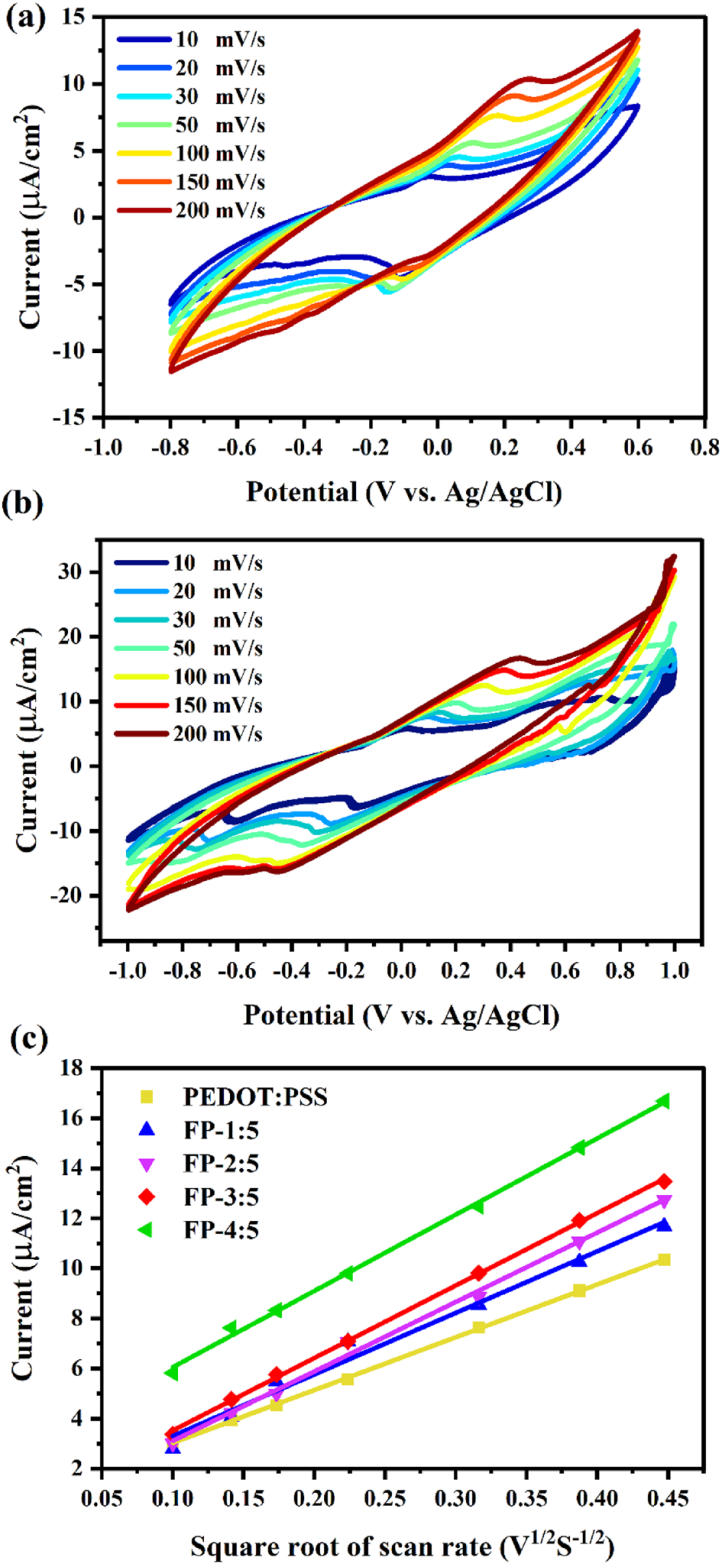
CV curves of (**a**) PEDOT:PSS, (**b**) FP-2:5 films at different scanning rates in a mixture solution of 10 mM K_3_(FeCN)_6_ and 0.1 M KCl and (**c**) plots of the oxidation current of [Fe(CN)_6_]^3−/4−^ (*Ip*) versus $v$^1/2^ with their corresponding fittings.

The active surface area of each film was compared using CV measurements based on 10 mM K_3_Fe(CN)_6_ and 0.1 M KCl solutions at various scanning rates. Figure 9a,b show the effect of scan rate ($v$) in the range from 10 to 200 mV/s on the CV response of PEDOT:PSS and FP-2:5 films. As expected, the peak current density varied linearly with increasing scan rates. Furthermore, using the Randles–Sevcik equation (Eq. 2)^53,54^, we obtained a linear dependence of the peak current as a function of the oxidation peaks of [Fe(CN)_6_]^3−/4−^ ($I_{p}$) versus the square root of the scan rate for these electrodes, as shown in Fig. 9c.

2 $$ I_{p} = 2.65 \times 10^{5} n^{3/2} AD^{1/2} cv^{1/2} $$

where $I_{p}$, $D$, $n$, $A$, and $c$ are the peak current, the diffusion coefficient of the species of interest (cm^2^/s^1^), the amount of electron in the reaction, the electrode area (cm^2^), and the concentration of species in the bulk solution (mol/L^1^), respectively. As seen in Fig. 9c, the composited films' oxidation slopes are higher than the PEDOT:PSS film, as listed in Table 1. These results suggest that the increase in the proportion of fly ash directly affected the increase in the active surface area of films. However, the ratio of fly ash to PEDOT:PSS that exceeds 2 to 5 g/mL results in an increase in the film's electrical resistivity, which can be confirmed by EIS technique.

**Figure 10:**
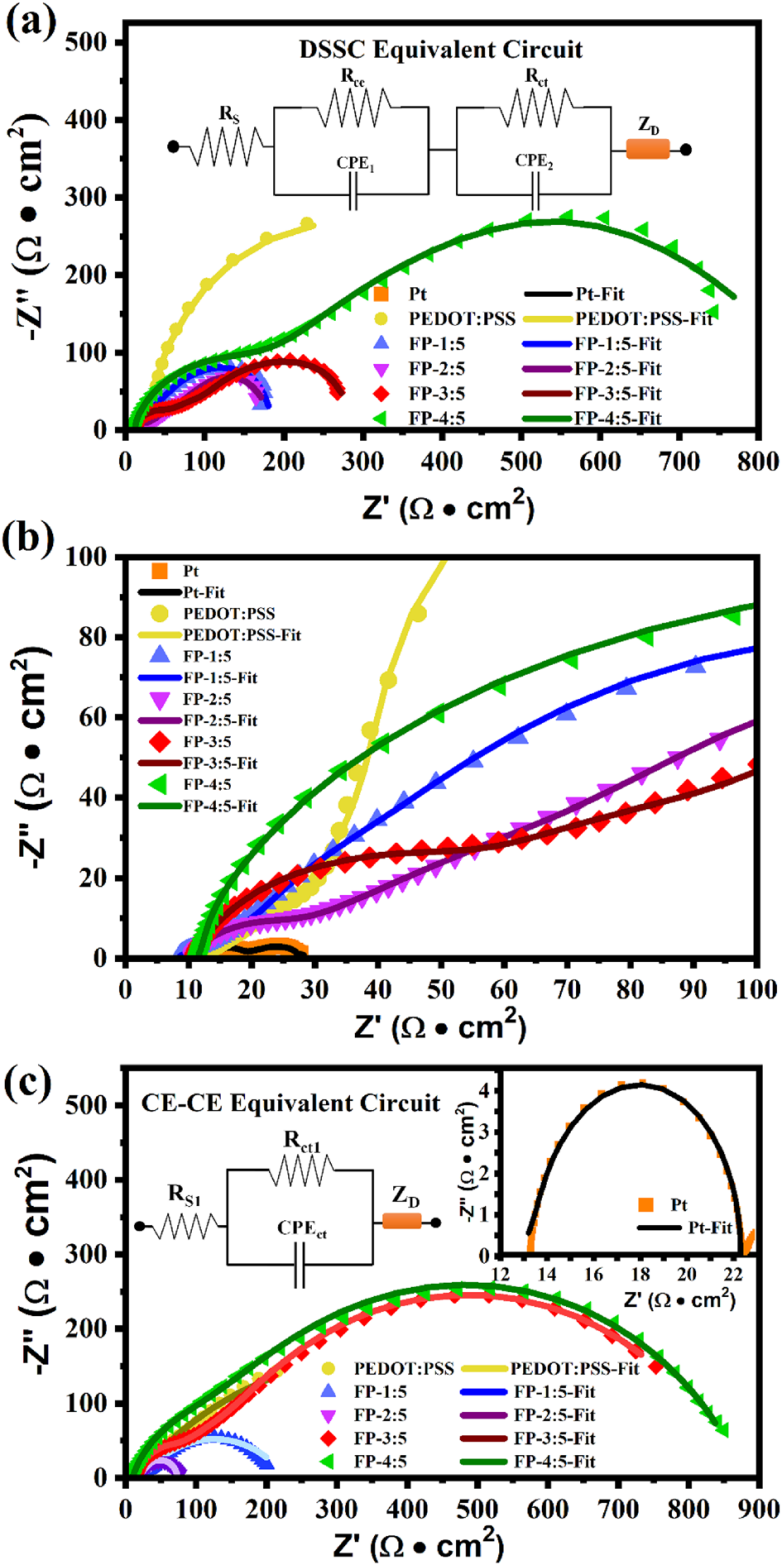
(**a**) Nyquist plots of the constructed DSSC cells and the inset DSSC equivalent circuit, (**b**) highly magnified Nyquist plots, and (**c**) Nyquist plots of the fabricated CE-CE cells (inset), the fabricated CE-CE equivalent circuit of prepared counter electrodes.

EIS measurements were accomplished with both DSSC asymmetric and CE-CE symmetric cells, as depicted in Fig. 10. This was done to evaluate the electrochemical characteristics of the prepared CEs. Figure 10a shows Nyquist plots of the prepared FP, PEDOT:PSS and Pt CEs. These plots can be fitted using Gamry Echem Analyst, to determine the facilitated electron transport in cells with equivalent circuits (inset in Fig. 10a) and the high-frequency range presented in Fig. 10b. In the Nyquist plot, $R_{s}$ is the total Ohmic resistance, which corresponds to the high frequency intercept of the real axis. The charge transfer resistance between the CE and electrolyte ($R_{ce}$), along with the TiO_2_/dye sensitizer and electrolyte ($R_{ct}$ or $R_{we}$) are represented by semicircular arcs in the high (the first semicircle) and medium (the second semicircle) frequency regions, respectively. The chemical capacitance ($CPE_{ce}$ and $CPE_{ct}$) at the electrode/electrolyte interface, as well as the Nernst diffusion impedance in the low frequency range (*Z*_*D*_)^55^ and the fitted EIS parameters are listed in Table 2. It is well established in the literature that low $R_{s}$, $R_{ce}$ and $R_{ct}$ values promote good charge transfer and high conductivity^4,54^.

**Table 2:** Summary of the electrochemical parameters of prepared samples.

Counter electrode	CE-CE cells		DSSC cells		
	$R_{s1}$ (Ωcm^2^)	$R_{ct1}$ (Ωcm^2^)	$R_{s}$ (Ωcm^2^)	$R_{ce}$ (Ωcm^2^)	$R_{ct}$ (Ωcm^2^)
Pt	13.30	9.14	13.25	4.09	10.27
PEDOT:PSS	8.13	732.00	6.99	31.24	280.50
FP-1:5	8.81	201.75	8.20	13.97	142.40
FP-2:5	9.93	64.30	10.27	12.06	91.45
FP-3:5	10.25	835.00	10.32	42.91	205.95
FP-4:5	7.91	865.00	11.61	157.45	732.50

The impedance of dye-sensitized solar cells ($Z_{DSSC}$) can be expressed as the sum of the impedance of Ohmic series resistance ($Z_{s}$), the impedance of charge transfer resistance at the counter electrode ($Z_{ce}$), the impedance of charge transfer resistance at the working electrode ($Z_{we}$), and the impedance of $\text{I}_{3}^{ - }$ and diffusion ($Z_{D}$) according to the following Eqs. (3–5)^56^.

3 $$ Z_{DSSC} = Z_{s} + Z_{ce} + Z_{we} + Z_{D} $$

The Eq. (3) can be written in term of resistances through the equation:

4 $$ Z_{DSSC} = R_{s} + \frac{{R_{ce} }}{{1 + \left( {j\omega } \right)^{\alpha } R_{ce} C_{ce} }} + \frac{{R_{we} }}{{1 + \left( {j\omega } \right)^{\alpha } R_{we} C_{we} }} + R_{D} \sqrt {\frac{{D/L^{2} }}{j\omega }} \tanh \sqrt {\frac{j\omega }{{D/L^{2} }}} $$

where $j$, $\omega$, $\alpha$, and $C_{ce}$ are the imaginary number ($j = \sqrt { - 1}$), the angular frequency, the exponent that equals 1 for a capacitor and is less than 1 for a constant phase element, and the electrochemical capacitance produced by the accumulation of surface electrons of a counter electrode film^57^. The $C_{we}$, $R_{D}$, $D$, and $L$ are chemical capacitance performed by the gathering of charges at the surface of the working electrode film, the diffusion resistance, diffusion coefficient of ${\text{I}}_{3}^{ - }$ and the effective thickness, respectively^28,52^.

In case of CE-CE symmetrical, the CE-CE impedance is given by

5 $$ Z_{CE - CE} = R_{s} + \frac{{2R_{ce} }}{{1 + \left( {j\omega } \right)^{\alpha } R_{ce} C_{ce} }} $$

The FP-2:5 CE has lower $R_{ce}$ and $R_{ct}$ values (12.06 and 91.45 Ωcm^2^) than FP-1:5 (13.97 and 142.40 Ωcm^2^), PEDOT:PSS (31.24 and 280.50 Ωcm^2^), FP-3:5 (42.91 and 205.95 Ωcm^2^), and FP-4:5 (157.45 and 732.50 Ωcm^2^). However, the Pt CE has the smallest $R_{ce}$ and $R_{ct}$ (4.09 and 10.27 Ωcm^2^), indicating better charge transfer and conductivity. A PEDOT:PSS CE has the smallest $R_{s}$ (6.99 Ωcm^2^), indicating a strong connection between the active film and the substrate. The $R_{s}$, $R_{ce}$, and $R_{ct}$ values of the prepared CEs present the same tend as in the case of the CE-CE symmetric cell, as shown in Fig. 10c. EIS parameters for various counter electrodes are listed in Table 2 after fitting with the CE-CE symmetric cell equivalent circuits shown in the inset of Fig. 10c using Gamry Echem Analyst. The series resistance is denoted as $R_{s1}$, the charge transfer resistance is represented as $R_{ct1}$ and the corresponding chemical capacitance at the CE electrode/electrolyte interface is *CPE*_*ct*_^11,58,59^. $R_{ct1}$ values of the FP-2:5 and Pt CEs were found to be 64.30 and 9.14 Ωcm^2^, respectively. The lower $R_{ct1}$ of the Pt CE can be attributed to its better charge transfer. Addition of fly ash to PEDOT:PSS in a ratio exceeding 2:5, resulted in $R_{ct1}$ values that tended to increase, presumably due to the poor conductivity of native fly ash. This results in low activity of the produced counter electrodes, as depicted in the CV analyses.

**Figure 11:**
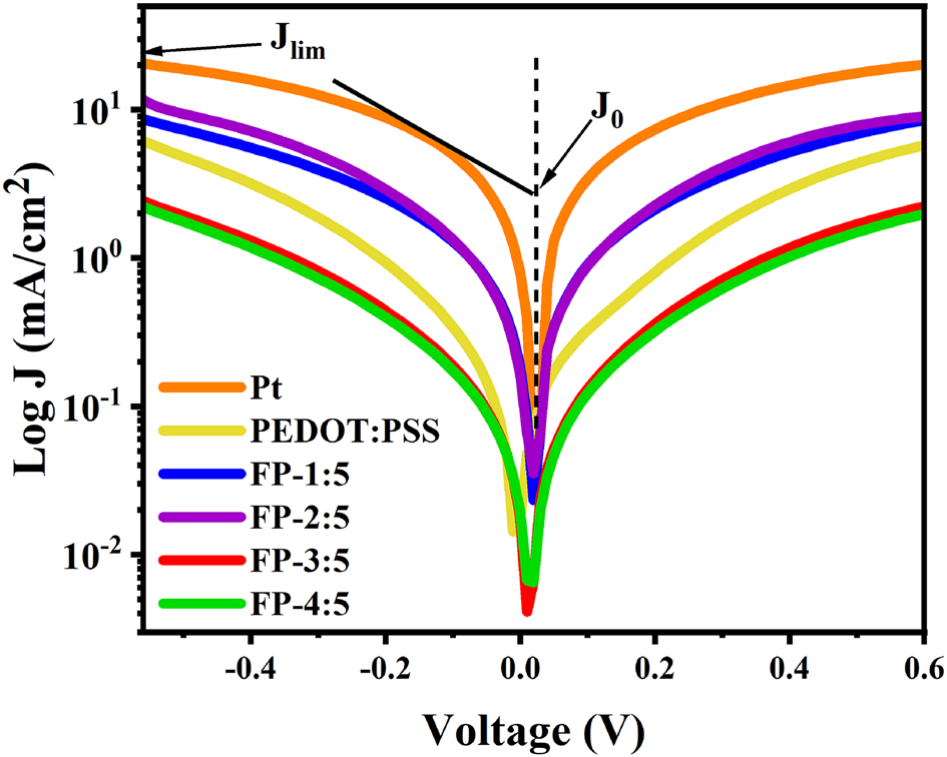
Tafel plots of the CE-CE cells of FP, Pt and PEDOT:PSS counter electrodes.

In Fig. 11, Tafel curves were also developed using a CE-CE cell similar to the one used in EIS. The exchange current density was estimated as the intersection of the extrapolated linear anodic and cathodic branches. These values varied in the order of Pt (1.60 mA/cm^2^) > FP-2:5 (0.38 mA/cm^2^) > FP-1:5 (0.30 mA/cm^2^) > PEDOT:PSS (0.11 mA/cm^2^) > FP-3:5 (0.05 mA/cm^2^) > FP-4:5 (0.04 mA/cm^2^). This is consistent with the variation trend of $R_{ct}$ according to the EIS results derived using Eq. (6). In the Tafel curves, the higher $J_{0}$ values correspond to lower $R_{ct}$ levels in EIS. The diffusion-limited current ($J_{\lim }$) values of the counter electrodes show that $J_{\lim }$ varies with the diffusion coefficient ($D$) depicted in Eq. (7). The resulting $J_{0}$ and $J_{\lim }$ values are presented in Table 1.

6 $$ J_{0} = \frac{RT}{{nFR_{ct} }} $$

7 $$ J_{\lim } = \frac{2nDCF}{l} $$

In the above equations, $R_{ct}$ and $R$ are the charge transfer resistances at CE/electrolyte interfaces and the gas constant, while $T$, $n$, $F$, and $l$ are the absolute temperature, the number of electrons involved in the reduction of triiodide at the electrode, Faraday’s constant, and the spacer thickness, respectively. $N_{A}$ is the Avogadro constant, and $C$ is the concentration of ${\text{I}}_{3}^{ - }$^49,60^. As can be seen, utilizing fly ash mixed with PEDOT:PSS in CEs increases the electrocatalytic activity of the electrodes compared to using PEDOT:PSS as the reference CE. Alternatively, the FP counter electrodes have lower electrocatalytic activity than Pt CE devices because of their higher $R_{ct}$ values. These results are well supported by CV and EIS analyses. For the purposes of lowering production costs, reducing toxicity in the environment and waste recycling, using fly ash is an attractive option to replace Pt metal in DSSCs.

**Figure 12:**
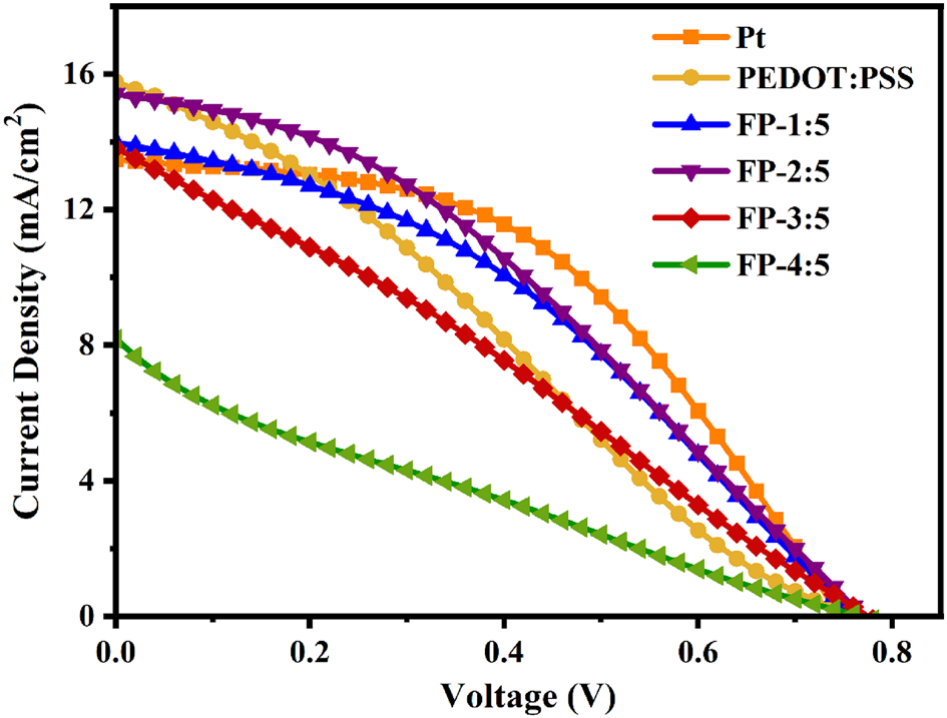
J-V curves of DSSCs assembled with various CEs under AM 1.5G (100 mW/cm^2^).

Figure 12 shows the J-V curves of the FP, PEDOT:PSS and Pt counter electrodes. Table 3 summarizes the photovoltaic parameters of these devices. The DSSC efficiency (PCE) was calculated according to the following Eqs. (8) and (9)^61^.

8 $$ {\text{PCE}} = \frac{{J_{sc} \times V_{oc} \times\text {FF}}}{{P_{in} }} \times 100\% $$

where $J_{sc}$, $V_{oc}$, and $P_{in}$ are the short-circuit current, the open-circuit voltage, and the input power, respectively. The fill factor (FF) of a DSSC can be estimated using the formula:

9 $$ \text{FF} = \frac{{P_{\max } }}{{J_{sc} \times V_{oc} }} $$

where, $P_{\max }$ is the maximum output power.

**Table 3:** A comparison of photovoltaic parameters of DSSCs assembled with various CEs, obtained at a 100 mW/cm^2^ (AM 1.5G) light intensity.

Counter electrode	$V_{oc}$ (V)	$J_{sc}$ (mA/m^2^)	$\text{FF}$	PCE (%)
Pt	0.741 ± 0.008	13.481 ± 0.469	0.481 ± 0.017	4.841 ± 0.151
PEDOT:PSS	0.761 ± 0.005	15.768 ± 0.622	0.279 ± 0.011	3.353 ± 0.271
FP-1:5	0.758 ± 0.001	13.984 ± 0.785	0.404 ± 0.052	4.097 ± 0.575
FP-2:5	0.769 ± 0.001	15.415 ± 0.960	0.357 ± 0.009	4.230 ± 0.225
FP-3:5	0.776 ± 0.002	13.819 ± 0.302	0.282 ± 0.009	3.022 ± 0.166
FP-4:5	0.768 ± 0.006	8.575 ± 0.465	0.218 ± 0.007	1.376 ± 0.125
MoO	0.760 ± 0.024	8.120 ± 1.160	0.500 ± 0.092	3.080 ± 0.136
CoO	0.760 ± 0.024	9.100 ± 0.895	0.520 ± 0.027	3.650 ± 0.246

The DSSC produced with a PEDOT:PSS based counter electrode had a PCE of 3.35% and FF of 0.28. The PCE value increased to 4.23% and the FF value improved to 0.36 when fly ash was mixed with PEDOT:PSS for a counter electrode with the ratio of fly ash to PEDOT:PSS of 2:5 g/mL. This was due to better charge transfer and higher electrocatalytic activity from the interaction of fly ash and PEDOT:PSS. Furthermore, as shown in Table 3 and Fig. S5, the prepared FP-2:5 counter electrodes had higher PCE values than those fabricated from other metal oxides, with PCE values of 3.08, and 3.65% for MoO and CoO, respectively. Due to the higher charge transfer and better electrocatalytic activity of the Pt CE, the PCE and FF values of the FP-2:5 CE are still lower than for the Pt CE (PCE of 4.84% and FF of 0.48). Generally, the FF of a DSSC is determined by two factors. The first is a DSSC's internal resistances, including both series resistance and charge transportation resistance. The greater the value of FF, the smaller the resistances. The second parameter is a DSSC counter electrode’s catalytic activity. The CV and EIS test results demonstrate that the platinum CE exhibits better electrocatalytic activity and higher charge transfer^62^, which results in higher FF and PCE values compared to the fly ash mixed PEDOT:PSS CEs. However, using fly ash mixed PEDOT:PSS as a possible replacement for Pt metal is a viable option because it decreases production costs, mitigates environmental impacts, and allows for waste recycling. In addition, it is well known that the CEs are important components in DSSCs whose stability tests are critical features for their practical applications. The research group of Yun et al*.*^52,63–67^ proposed technique for effectively testing the stability of the CEs. For example, they conducted successive CV scanning, EIS scanning, long-term stability, under dark and illumination current–voltage test, removal rate of the films and so on. These measurements can used to very well verify the stability of the CEs in DSSCs.

## Conclusions

In this paper, we introduced novel fly ash mixed PEDOT:PSS counter electrodes that can be used to fabricate low-cost DSSCs. We found that adding fly ash to PEDOT:PSS improved the electrocatalytic activity, carrier transport and photovoltaic properties of the counter electrodes. A greater PCE, 4.23%, was obtained using an appropriate ratio, 2:5 g/mL, of fly ash to PEDOT:PSS. Furthermore, the FP-2:5 counter electrode outperformed MoO and CoO counter electrodes in DSSCs. Alternatively, the FP-2:5 counter electrode had a greater triiodide reduction rate than a Pt CE. As a result, FP-2:5 is an intriguing alternative material for manufacturing inexpensive and ecologically friendly solar cells employing waste from coal power plants in the place of expensive Pt metal. Moreover, fly ash from coal power plants may be mixed with other catalyst polymers (such as PANI and PPY) to develop solar cell and for other applications (such as supercapacitors, fuel cells, batteries and splitting water molecules) because of the high effectiveness of this material.

## Supplementary Information

Supplementary Figures.

## Data Availability

All data generated or analyzed during this study are included in this published article [and its supplementary information files].
