# Supplementary material for: Fly ash boosted electrocatalytic properties of PEDOT:PSS counter electrodes for the triiodide reduction in dye-sensitized solar cells
Source: Sci Rep. 2023 Apr 12;13:6012. doi: 10.1038/s41598-023-33020-6 (PMC10097718; doi:10.1038/s41598-023-33020-6)
Supplement: Supplementary file 1 — Supplementary Figures. [file 41598_2023_33020_MOESM1_ESM.docx]

**Supplementary Information**

**Fly ash boosted electrocatalytic properties of PEDOT:PSS counter electrodes for the triiodide reduction in dye-sensitized solar cells**

Nattakan Kanjana^a^, Wasan Maiaugree^a,b,^*, Paveena Laokul^c^, Inthira Chaiya^d^ ,Thodsaphon Lunnoo^a,b^, Poramed Wongjom^b^, Yingyot Infahsaeng^b,e^, Bunjong Thongdang^f^ and Vittaya Amornkitbamrung^g^

^a^Thammasat University Research Unit in Energy Innovations and Modern Physics (EIMP), Thammasat University, Pathum Thani 12120, Thailand

^b^Division of Physics, Faculty of Science and Technology, Thammasat University, Pathum Thani 12120, Thailand

^c^Department of Physics, Faculty of Science, Mahasarakham University, Kantarawichai, Mahasarakham 44150, Thailand

^d^Department of Mathematics, Faculty of Science, Mahasarakham University, Kantarawichai, Mahasarakham 44150, Thailand

^e^Thammasat University Research Unit in Quantum Technology Thammasat University, Pathum Thani 12120, Thailand

^f^Ratchaburi Electricity Generating Co. Ltd. Ratchaburi 70130 Thailand

^g^Thailand Center of Excellence in Physics, Commission on Higher Education, Bangkok 10400, Thailand

^*^Corresponding author: Tel.: +66862345510

E-mail address: wasankim@tu.ac.th
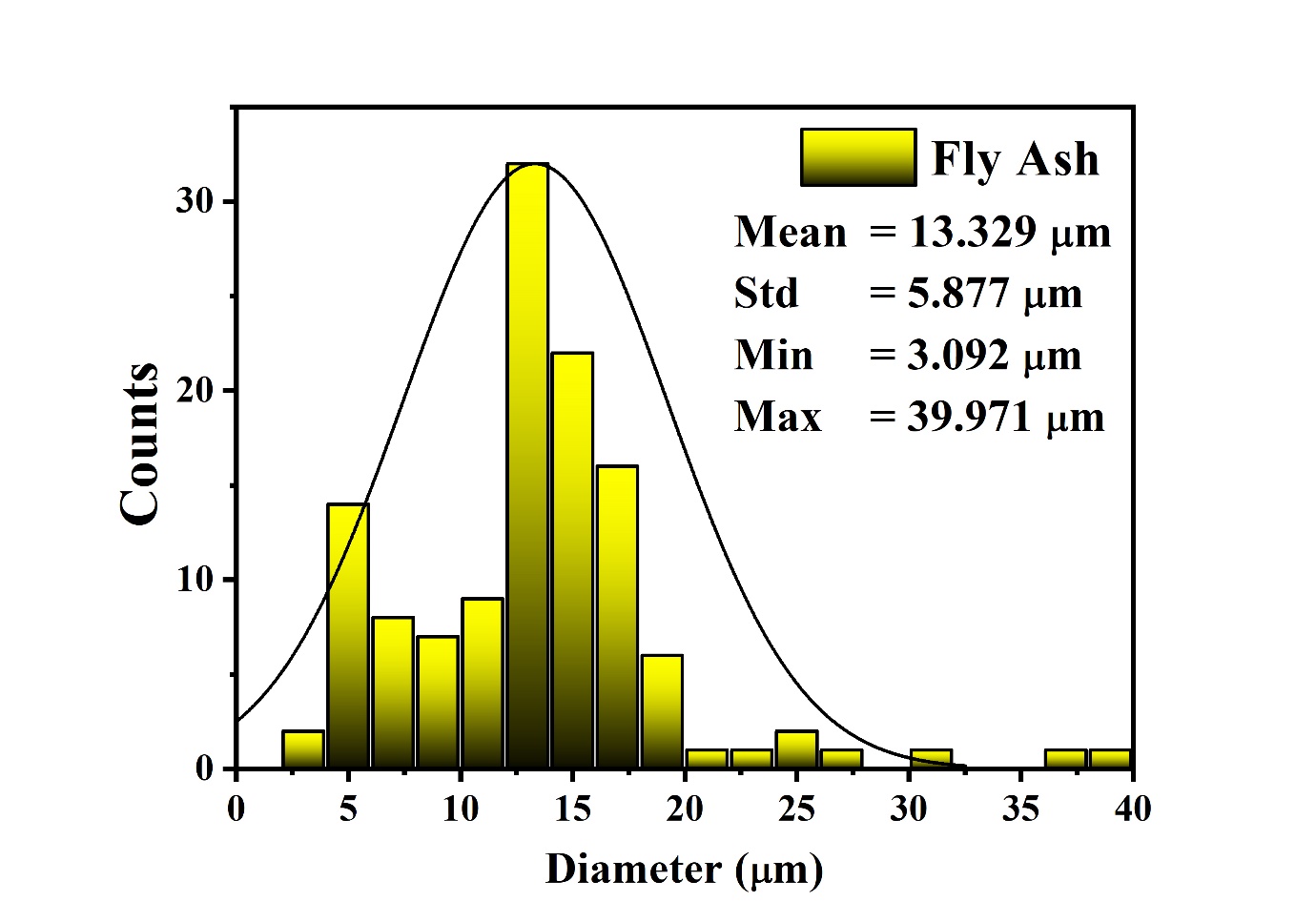


**Figure S1.** Particle diameter distribution of fly ash.


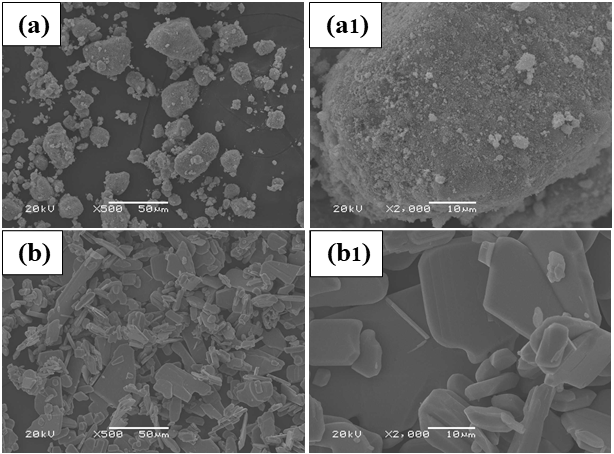


**Figure S2.** SEM images of (a and a1) CoO, and (b and b1) MoO powders.


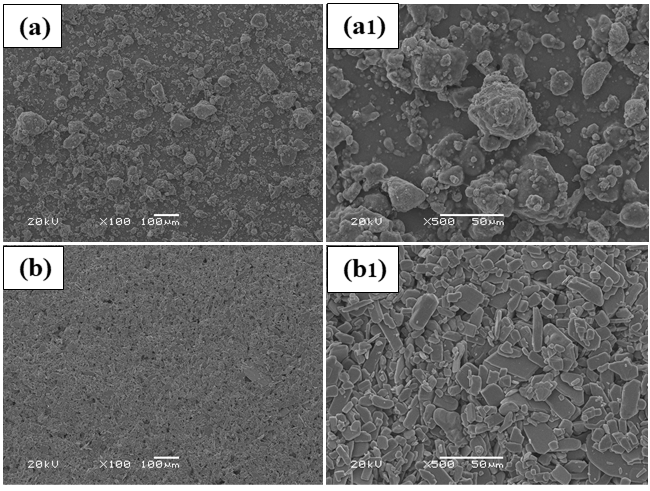


**Figure S3.** SEM images of (a and a1) CoO, and (b and b1) MoO films.

**Figure S4**. Cumulative intrusion curves and pore size distribution curves of the fly ash powder (figure inset).

**Figure S5.** J-V curves of DSSCs assembled using various CEs under AM 1.5G (100 mW cm^‑2^) of FA0.2g, Pt, CoO, and MoO CEs.
